# Cardiac aging synthesis from cross-sectional data with conditional generative adversarial networks

**DOI:** 10.3389/fcvm.2022.983091

**Published:** 2022-09-23

**Authors:** Víctor M. Campello, Tian Xia, Xiao Liu, Pedro Sanchez, Carlos Martín-Isla, Steffen E. Petersen, Santi Seguí, Sotirios A. Tsaftaris, Karim Lekadir

**Affiliations:** ^1^Artificial Intelligence in Medicine Lab (BCN-AIM), Universitat de Barcelona, Barcelona, Spain; ^2^Institute for Digital Communications, School of Engineering, University of Edinburgh, Edinburgh, United Kingdom; ^3^William Harvey Research Institute, NIHR Barts Biomedical Research Centre, Queen Mary University London, London, United Kingdom; ^4^Barts Heart Centre, St Bartholomew's Hospital, Barts Health NHS Trust, London, United Kingdom; ^5^Health Data Research UK, London, United Kingdom; ^6^The Alan Turing Institute, London, United Kingdom

**Keywords:** aging heart, generative adversarial network, magnetic resonance imaging, synthesis, data augmentation

## Abstract

Age has important implications for health, and understanding how age manifests in the human body is the first step for a potential intervention. This becomes especially important for cardiac health, since age is the main risk factor for development of cardiovascular disease. Data-driven modeling of age progression has been conducted successfully in diverse applications such as face or brain aging. While longitudinal data is the preferred option for training deep learning models, collecting such a dataset is usually very costly, especially in medical imaging. In this work, a conditional generative adversarial network is proposed to synthesize older and younger versions of a heart scan by using only cross-sectional data. We train our model with more than 14,000 different scans from the UK Biobank. The induced modifications focused mainly on the interventricular septum and the aorta, which is consistent with the existing literature in cardiac aging. We evaluate the results by measuring image quality, the mean absolute error for predicted age using a pre-trained regressor, and demonstrate the application of synthetic data for counter-balancing biased datasets. The results suggest that the proposed approach is able to model realistic changes in the heart using only cross-sectional data and that these data can be used to correct age bias in a dataset.

## Introduction

Understanding the effects of the aging process is becoming more important as the life expectancy increases worldwide. Aging has crucial implications for health and age is the main risk factor for the development of cardiovascular disease (1, 2). Insights into the aging mechanism can be very valuable to inform new interventions to delay the occurrence of possible adverse events and for improving health of the elderly.

According to the medical literature, age is positively related to morphological changes in the heart such as increased left atrial diameter (3), increased wall thickness in the left ventricle (LV) and reduced LV dimensions (1, 2). These changes are associated with atrial fibrillation and heart failure with preserved ejection fraction (2, 4). Females and males show differences in the aforementioned changes with increased LV wall thickness being more prevalent in women (5). Also, a marked increase in epicardial adipose tissue deposition has been observed with age (5).

Collecting longitudinal data is very time-consuming and requires repeated visits of participants with the associate chance of dropouts along the duration of the study. Two longitudinal studies have analyzed cardiac health with more than three decades of measurements. These are the Framingham Heart Study (FHS) (6) and the Baltimore Longitudinal Study on Aging (7). However, imaging is only available for the FHS and only for echocardiography. Imaging with higher spatial resolution may be found in two other longitudinal studies, the UK Biobank (ukbiobank.ac.uk) and the Multi-Ethnic Study of Atherosclerosis (MESA) study (8), where participants are scanned using magnetic resonance imaging (MRI). However, only a subset of participants have repeated scans adquired in the next 1–10 years after the first scan visit. Thus, modeling the aging process in the heart with good spatial resolution is restricted to 10 years or less if one relies only on longitudinal data, and the analysis may be limited by small differences in the patient positioning between visits. A potential data-driven approach, however, that leverages cross-sectional data, i.e., data from different participants with different age, to synthetically age or rejuvenate a real image could boost the efficient use of such a large cohort. In recent years, models based on generative adversarial networks (GANs) (9) have been proposed for this task.

Deep learning models for synthesizing an aged version of an input image have been proposed for several applications, but especially for face aging. For example, Zhang et al. (10) was one of the first works to propose learning a manifold of images, *via* cross-sectional data, that can be navigated for increasing or decreasing the apparent age of a human face. The authors used an autoencoder and adversarial training to generate photorealistic images of a younger and older version of an input face. Later, Liu et al. (11) used a GAN-based model that included also attribute conditioning such as race or sex to enforce attribute preservation, highlighting the importance of covariates for the modeling. Contrary to Zhang et al. (10), their model had a last layer responsible for fusing the input image with the generated features, so that the model did not need to generate the whole image as output.

In medical imaging, a recent study by Xia et al. (12) proposed a conditional GAN (cGAN) (13) that considered age and disease status for generating an aged brain MRI using only cross-sectional data. Other works have modeled the changes in the brain due to aging with autoencoders and adversarial training (14, 15) or with normalizing flows (16), although the image quality was worse in these cases. Finally, cGANs have also been applied recently to synthesize future fundus images given a lession probability map and a vessel segmentation (17).

In this work, we propose a conditional generative model for extracting longitudinal patterns related to aging from cross-sectional data and apply it to cardiac imaging for the first time, to the best of our knowledge. Moreover, we demonstrate the model applicability for counter-balancing biased datasets with respect to age. Finally, we analyze the modeling ability of the proposed approach for two other tasks: apparent body mass index modification and end-systolic phase synthesis from end-systolic frames.

## Materials and methods

### Dataset

For this work, MRI studies from the UK Biobank were used. These studies contain short- and long-axis views of 43,352 participants (including 23,508 female subjects). The participants were scanned at ages between 45 and 82 years old (mean age 64.1 ± 7.7). The scanner used was a MAGNETOM Aera, syngo MR D13A (Siemens, Erlangen, Germany) with a field strength of 1.5 Tesla [see (18) for further details about the imaging protocol]. Only the four chamber view was used in this work for simplicity and in order to include information from all heart chambers during the modeling. The end-diastolic phase for each subject was identified and used in this work, given that the model was two-dimensional. A total of 14,788 subjects were selected for training the generative models. No preprocessing was applied to the images.

Additionally, 764 ground-truth annotations of the four chamber long axis view performed by expert cardiologists from the Barts Heart Centre were made available to the authors from a previous work (19). The regions of interest annotated were the left and right ventricular cavities, the left ventricular myocardium and the left and right atria. We also delineated the aorta in 50 samples. Automatic segmentations for the rest of participants were generated for the four chambers, the myocardium and the aorta by training a U-Net model (20) (details in Supplementary material).

### Conditional generative modeling

**Figure 1:**
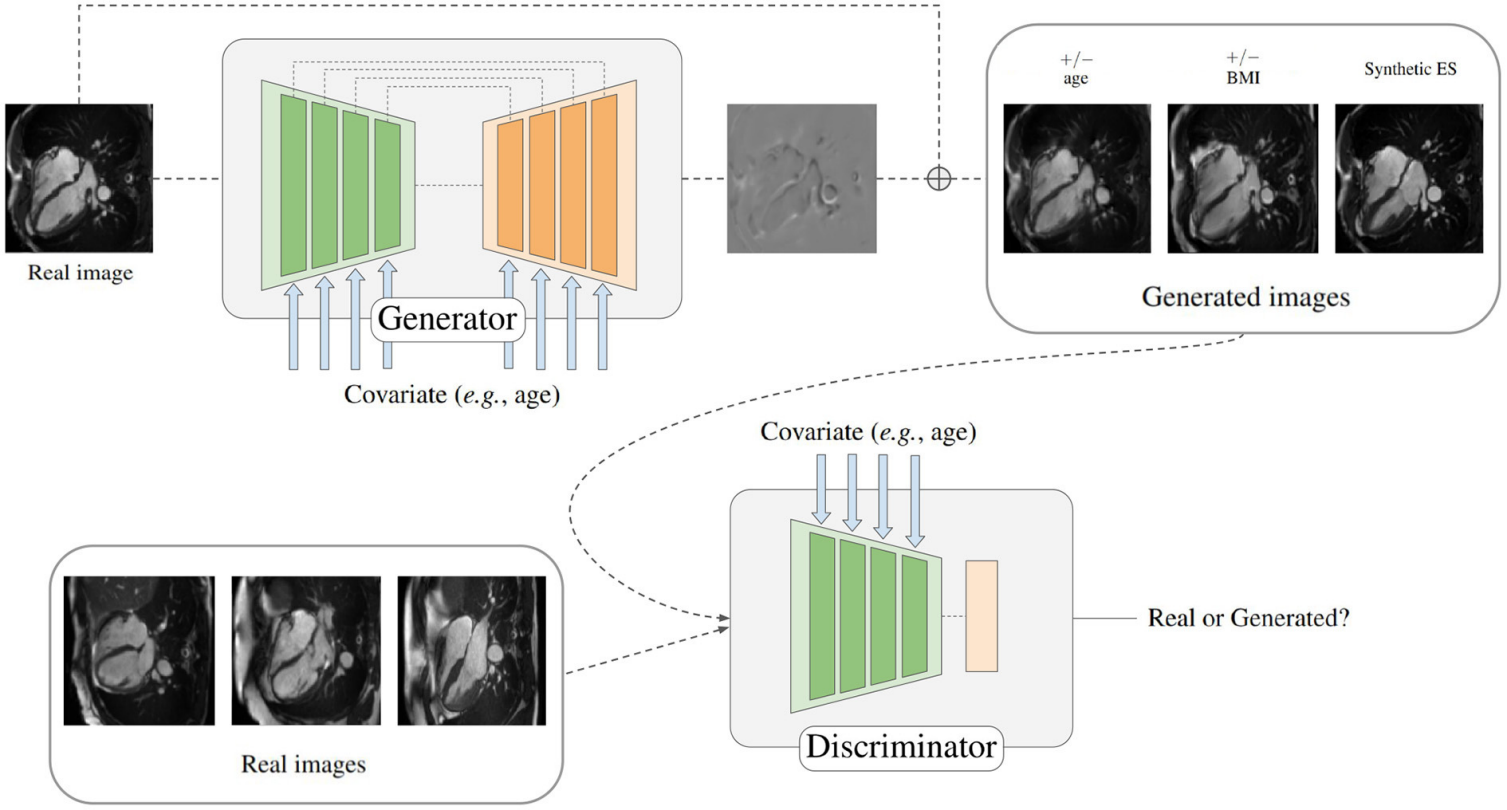
Depiction of the proposed model for generating synthetically aged and rejuvenated heart images. The covariate is combined with the model features by using conditional biasing. Heart scans reproduced by kind permission of UK Biobank ©.

In order to generate synthetic images of the heart depending on a given covariate, a conditional generative adversarial network is proposed, as depicted in Figure 1. The two components of the model are a generator and a discriminator. Specifically, the generator is responsible for creating the mapping that will be applied to the input image when conditioned on different covariates to obtain a target image, while the discriminator is trained to tell apart real and synthesized images given some covariates.

**Figure 2:**
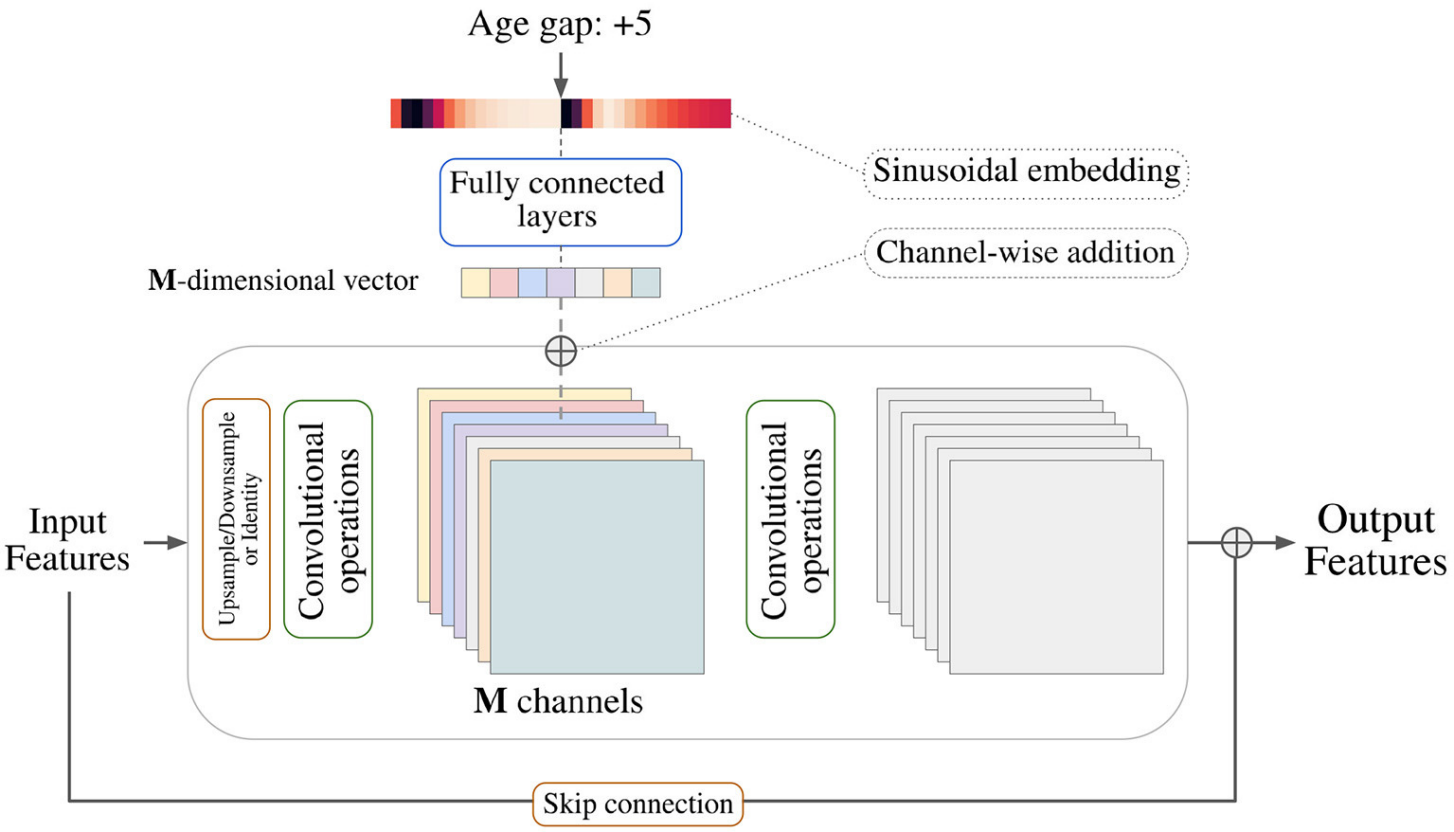
Depiction of a general residual block and the conditional biasing mechanism used to pass covariates to the model. A sinusoidal embedding is applied to transform the scalar covariate into a vector that is later converted to a vector with the same dimensionality as the number of intermediate feature channels. Each value in the vector is then added to its corresponding channel in order.

The architecture of the generator follows a typical U-Net (20) encoding-decoding scheme where each layer is composed of stacks of two residual blocks with an intermediate attention block. It is based on the generator used in recent state-of-the-art diffusion models (21, 22) that was first introduced by Ho et al. (23). The discriminator consists of an encoder, just as the one used for the generator, and an adaptive pooling layer. Each residual block along the networks is conditioned on the input variable by using *conditional biasing*, i.e., by transforming the variable into a vector of varying dimension and adding one value per intermediate feature channel prior to a group normalization step. Figure 2 depicts the conditional biasing mechanism in more detail. This type of conditioning allows for a better conservation of the input information throughout the network by consistently introducing the conditional variable on each layer. Additionally, the network is able to fit the different parameters used to compute the conditioning vector on each layer separately, enhancing the ability of the model to learn different distributions at different resolutions.

As covariates, age and body mass index (BMI) were considered for two separate tasks. The generator was conditioned with the difference between the age (respectively BMI) of the input image and the desired output age (respectively BMI). The discriminator, however, was conditioned on the actual age (respectively BMI) of the input image (real or synthesized). The covariate was specified to the model using the Transformer sinusoidal embedding (24).

### Training details

The underlying framework for training the model relied on the Wasserstein-GAN with a gradient penalty term (WGAN-GP) (25, 26), that achieved better results than usual GANs, by minimizing the Wasserstein-1 distance (also called Earth-Mover distance).

The generator (*G*) and the discriminator (*D*) were trained using the adversarial objective loss for WGAN-GP:


(1) $$\begin{array}{l} \begin{array}{l} L_{\text{WGAN-GP}}={𝔼}_{\tilde{\text{x}}\sim P_{gen.}}\left[D\left(\tilde{\text{x}},{\text{a}}_{t}\right)\right]-{𝔼}_{\text{x}\sim P_{real}}\left[D\left(\text{x},{\text{a}}_{t}\right)\right]\\ \;+\lambda_{GP}{𝔼}_{\hat{\text{x}}\sim P_{\hat{\text{x}}}}\left[{\left(||\nabla_{\hat{\text{x}}}^{2}D\left(\hat{\text{x}},{\text{a}}_{t}\right)||-1\right)}^{2}\right], \end{array} \end{array}$$

where **a**_*s*_ and **a**_*t*_ stand for source and target age, respectively, **x** is the input image, $\tilde{\text{x}}=G\left(\text{x},{\text{a}}_{d}\right)$ is the generated sample with age gap **a**_*d*_ = **a**_*t*_ − **a**_*s*_, and $\hat{\text{x}}=\epsilon \text{x}+\left(1-\epsilon \right)\tilde{\text{x}}$, with ϵ~U(0,1), is a random point along the line connecting the real and generated samples. *P*_*real*_ and *P*_*gen*_ represent the distributions of real and generated images, respectively. The gradient penalty factor, λ_*GP*_, was set to 10 in all experiments following the original work by Gulrajani et al. (26).

In addition to the adversarial loss, a cycle-consistency term was considered to enforce the reconstruction of the original image after two generator steps, one for aging (rejuvenating) and one for rejuvenating (aging) the subject back to the original state. In detail, the difference between the transformed image (after adding **a**_*d*_ years) and the reconstructed image (after subtracting **a**_*d*_ years to the transformed image) was minimized. This term is formally written as


(2) $$\begin{array}{l} L_{cc}=\underset{\text{x}\sim P_{real}}{𝔼}\left[||\text{x}-G\left(G\left(\text{x},{\text{a}}_{d}\right),-{\text{a}}_{d}\right){||}_{1}\right]. \end{array}$$

Overall, the final objective loss was


(3) $$\begin{array}{l} L=\underset{G}{\min}\underset{D}{\max}\left(L_{\text{WGAN-GP}}+\lambda_{cc}L_{cc}\right), \end{array}$$

where the weight λ_*cc*_ was empirically set to 1 based on model performance.

During training, WGAN-GP requires the discriminator performance to be close to optimal. For this reason, the first 20 epochs were used as a warm-up period, and the discriminator was updated 50 times for every generator update. For the remaining epochs, the discriminator was updated five times for every generator update. The AdamW (27) optimizer was used for both networks with a learning rate and weight decay of 10^−4^ and first and second moments equal to 0.9 and 0.999, respectively. Data augmentation was used to increase the variability in the input images appearance. The transformations considered were random bias field addition, random histogram shift and random contrast adjustment (28). The images were cropped along the *x* axis by 90 pixels, resized to 128^2^ pixel size and the intensities rescaled to the [0, 1] range. The generated mapping is an array of shape 128^2^ with values clipped to the range [−1, 1] (the maximum modification allowed to the input image). After the addition of the mapping, the resulting image was again clipped to [0, 1]. The whole training process took ~90 h in a Nvidia 3090 GPU for 300 epochs and with a batch size of 12 images. PyTorch (version 1.10.0) was used for the implementation.

## Results

Given the lack of real longitudinal data with a time span between visits larger than 10 years and the added factor of morphological variations attributed to different patient positioning, we propose to evaluate the current model using two proxy approaches that circumvent the limited time span and the potential disalignment between scans. First, we assess the resulting synthetic images *via* age accuracy and image quality, and compare the proposed model against two baselines. Second, we train age regressors with an imbalanced dataset augmented with synthetic samples. Moreover, in order to demonstrate the modeling capabilities of the current approach, two alternative tasks with an easier interpretation are considered: (1) BMI modification and (2) end-diastolic to end-systolic phase transformation.

### Qualitative results

**Figure 3:**
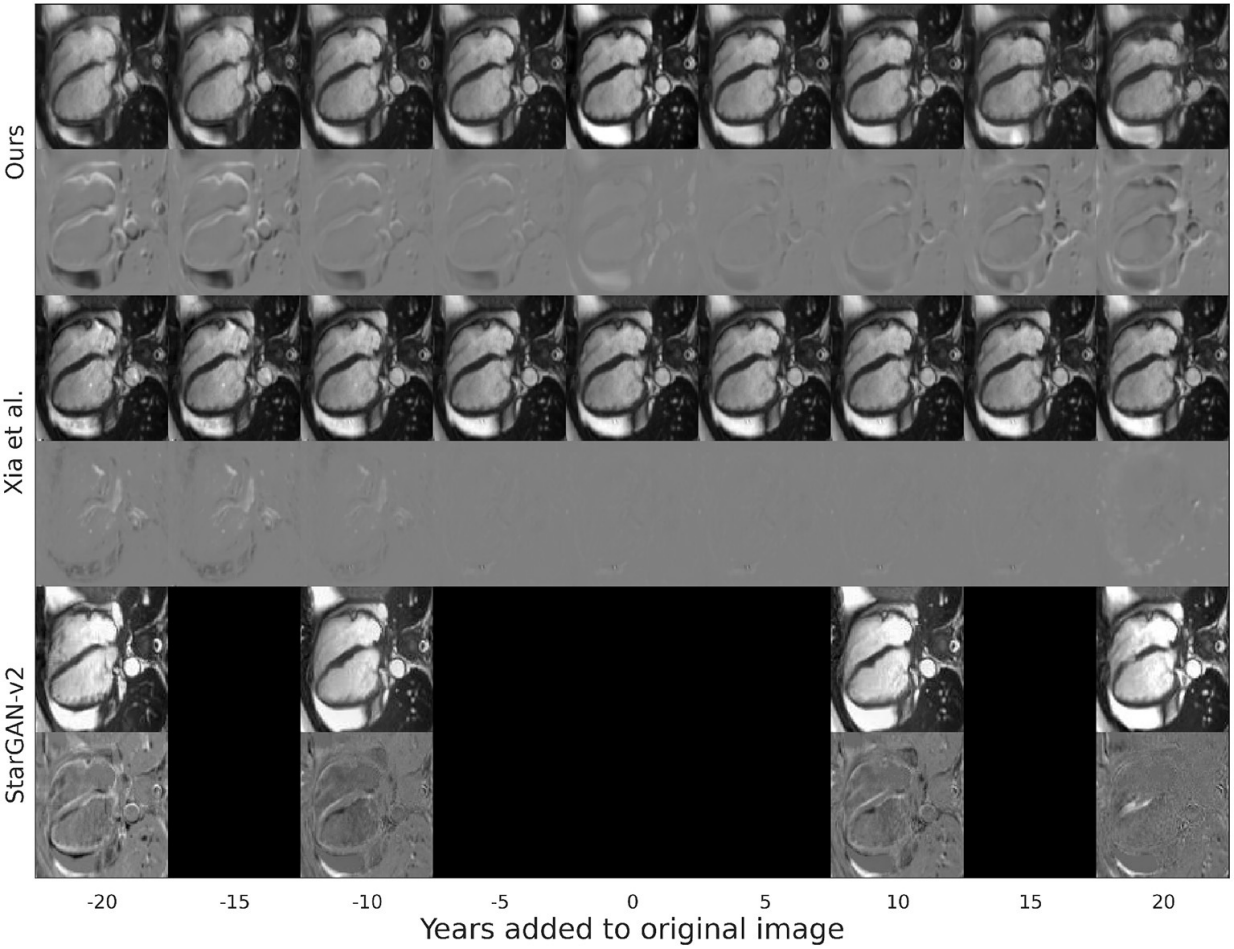
Synthetic aged and rejuvenated images for a randomly selected subject from the test set for the current proposal and the two baselines. Each pair of rows contain the generated image and the mapping applied to the original image to obtain it. The column with age gap equal to zero represents the reconstructed image. Reproduced by kind permission of UK Biobank ©.

At a qualitative level, as presented in Figure 3, the changes of our proposal tend to be more localized in space than the modifications introduced by the other baseline models. These modifications focus mostly on the interventricular septum and the aorta with opposed transformations for opposite age gaps. In detail, for increased age, the interventricular septum is enlarged toward the LV cavity and the aorta is enlarged. Finally, although most of the changes occur in the heart, some modifications are observed in surrounding areas.

### Quantitative assessment of generated images

#### Assessment *via* predicted age

The apparent age of synthesized images was assessed using a pre-trained ResNet18 (29) age regressor (MAE: 4.6 ± 3.2 years for males and 3.9 ± 3.1 years for females). The hypothesis was that images aged (respectively rejuvenated) by the model should have a target age greater (respectively lower) than the original images. Tables 1, 3 show the mean absolute error (MAE) for age predictions using the pre-trained regressor when tested on the images generated by the proposed approach and compare it to a model adapted from the work of Xia et al. (12) that generates images with a controllable age gap and to StarGAN-v2 (30) that transforms images between fixed age gaps (gaps of 10 and 20 years), respectively.

**Table 1:** Mean absolute error (MAE) for age prediction of images generated synthetically from a random testing set of 907 subjects with varied age and grouped in age gaps of 5 years.

	**MAE for predicted age (**|**predicted age** −**target age**|**)** ↓							
**Age gap**	**−20**	**−15**	**−10**	**−5**	**5**	**10**	**15**	**20**
Ours	7.2_4.3_	5.8_3.8_	6.0_4.0_	4.7_3.6_	4.8_3.4_	5.8_3.8_	5.8_3.7_	6.2_3.6_
Xia et al. (12)	12.8_4.7_	9.4_4.8_	6.8_4.4_	5.7_4.2_	5.7_3.9_	8.7_4.7_	10.6_4.9_	12.8_5.0_
Zero order	16.4_4.3_	12.6_4.7_	8.7_4.7_	5.6_4.1_	6.0_4.1_	9.2_4.8_	12.8_4.9_	16.6_4.7_

Results are presented for models with the ability to generate a variable age gap: our proposal and the adapted model by Xia et al. (12). The Zero order shows the prediction error for unmodified images. The best results are shown in bold face and standard deviations as subscripts.

The last row (Zero order) corresponds to the results obtained when the original images are not modified at all, i.e., the predicted age is always the same but the target age changes according to the desired age gap. We find that the proposed model with residual and attentional blocks outperforms the model based on the work by Xia et al. (12) and it obtains comparable results to StarGAN-v2, while StarGAN-v2 can only translate images between fixed domains. The proposed approach presents a significant improvement in MAE when compared to the Zero order.

#### Assessment *via* image quality

Image quality was assessed *via* the Fréchet inception distance (FID) (31) and the peak signal-to-noise ratio (PSNR). The FID gives a sense of how different two datasets are in terms of features extracted from a pre-trained deep learning model (better quality corresponds to lower values). An InceptionV3 model (32) was trained on the UK Biobank for this purpose (further details in Supplementary material). PSNR, on the other hand, evaluates the amount of corruption or noise in the generated images by directly comparing them to the original ones (better quality corresponds to higher values). As observed in Table 2, both metrics were coherent and showed better image quality for the model based on the work on Xia et al. (12), while StarGAN-v2 obtained images with significantly worse image quality (see Table 3).

**Table 2:** Image quality in terms of Fréchet inception distance (FID) and peak signal-to-noise ratio (PSNR) for images generated synthetically from a random testing set of 907 subjects with varied age and grouped in age gaps of 5 years.

	**FID** ↓								**PSNR (dB)** ↑							
**Age gap**	**−20**	**−15**	**−10**	**−5**	**5**	**10**	**15**	**20**	**−20**	**−15**	**−10**	**−5**	**5**	**10**	**15**	**20**
Ours	1.0	1.0	1.0	0.9	1.1	1.2	1.8	2.2	19.7	21.2	24.4	25.9	26.6	25.0	21.2	19.2
Xia et al. (12)	0.4	0.4	0.2	0.0	0.0	0.0	0.2	0.5	28.0	28.4	30.7	49.8	63.8	58.7	44.0	31.2

Results are presented for models with the ability to generate a variable age gap: our proposal and the adapted model by Xia et al. (12).

**Table 3:** Mean absolute error (MAE) for age prediction and image quality metrics (FID and PSNR) for synthetically generated images from a subset of subjects with 60 and 70 years old (for an age gap of 10) and with 55 and 75 years old (for an age gap of 20).

	**MAE for predicted age** ↓				**FID** ↓				**PSNR (dB)** ↑			
**Age gap**	**−20**	**−10**	**10**	**20**	**−20**	**−10**	**10**	**20**	**−20**	**−10**	**10**	**20**
Ours	4.7_4.1_	5.0_3.1_	4.1_3.3_	5.4_3.0_	0.9	1.2	1.2	1.5	19.4	24.5	25.0	19.1
Xia et al. (12)	10.8_4.1_	5.6_3.2_	7.8_4.4_	12.6_4.6_	0.4	0.2	0.0	0.5	28.1	29.7	58.8	30.9
StarGAN-v2	4.2_4.3_	5.2_3.0_	9.2_4.0_	5.1_4.7_	12.5	5.0	12.4	9.9	9.8	10.3	10.0	9.7
Zero order	16.4_4.3_	8.7_4.7_	9.2_4.8_	16.6_4.7_	–	–	–	–	–	–	–	–

Best results are shown in bold face and standard deviations as subscripts.

This can be attributed to Xia et al.'s model introducing less modifications in the image (see Figure 3), resulting in synthetic images that are more similar to the original images but that do not represent the target age accurately as shown when computing the predicted age error in Table 1. On the other hand, StarGAN-v2 is introducing more modifications in the image, degrading its quality (see Figure 3), while maintaining a competitive predicted age error in Table 3.

### Volumetric analysis

**Figure 4:**
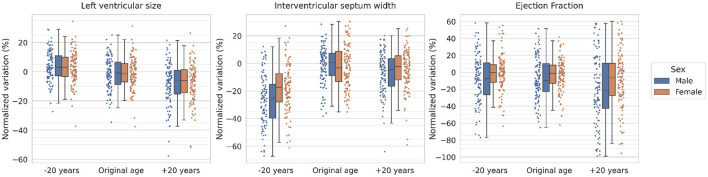
Normalized volumetric variations of synthesized images for the left ventricle, the interventricular septum and the ejection fraction. Every dot represents a subject and the boxes represent the interquartile range. The normalized variation is computed as the normalized difference of the selected metric with respect to the mean value for the original distribution (i.e., the “Original age” distribution). The results are obtained for a subset of participants aged 60.

In order to quantify the specific changes performed in the heart, the LV size, the interventricular septum width and the ejection fraction are derived from automatically generated segmentations of the original and synthesized images (more details about the segmentation model are provided in Supplementary material). The normalized variation for these metrics after adding or subtracting 20 years to the original subjects is presented in Figure 4 separated by sex.

As observed in the figure, the model shows a clear tendency for decreased LV size with age, going from a 5% increase for rejuvenated subjects to a 5% decrease for aged subjects (with respect to the original sample) for both sexes. With respect to the interventricular septum average width, a decrease around 25% for males and 15% for females is observed for rejuvenated images, while the aged images also show a decrease of around 5–10% for both sexes. Finally, the ejection fraction shows a similar distribution for rejuvenated and original images while the aged subjects present a larger variability and an overall mean decrease of around 20% for males and 5% for females.

### Synthetic images as data augmentation

Finally, in order to assess the utility of generated images for data augmentation, several age regressors (ResNet18) were trained with two datasets created from a new sample of 1,000 subjects with a particular age imbalance. Dataset one (D1) consisted of an imbalanced dataset with 90% of subjects younger than 70 years old. Dataset two (D2) was constructed to manifest an imbalance for younger patients, with 90% of the subjects being older than 60 years. These datasets were gradually augmented with 1, 5, 10, and 25% of synthetically aged (for D1) or rejuvenated (for D2) subjects. The results are presented in Table 4. A clear reduction in prediction error is observed when using synthetically age (or rejuvenated) subjects and the error when using 10 or 25% of synthetic images is comparable to the error obtained with a balanced dataset (12.7 ± 8.9).

**Table 4:** Mean absolute error (MAE) of ResNet18 age regressors when trained with two imbalanced datasets and different proportions of added synthetic images.

	**MAE** ↓				
	**0%**	**1%**	**5%**	**10%**	**25%**
D1 (10% of older subjects)	14.5_9.0_	13.3_8.6_	14.2_9.3_	12.8_8.6_	11.0_7.6_
D2 (10% of younger subjects)	18.0_9.7_	17.0_9.7_	17.8_10.0_	15.4_9.4_	13.9_9.2_
Balanced dataset	12.7_8.9_	–	–	–	–

Standard deviations are presented as subscripts.

### Alternative tasks

In order to showcase the capabilities of the proposed approach, the same model is used for modifying the BMI of an input patient and for transforming an image in the end-diastole (ED) time frame to an image in end-systole (ES).

Table 5 compares the prediction error (MAE) between apparent BMI and the target BMI, as obtained from a pre-trained ResNet18 BMI regressor (MAE 1.4 ± 1.1 for males and 1.6 ± 1.4 for females), for images generated with the proposed model and for images that were not modified at all (Zero order). The MAE increases slightly with higher BMI differences between input and synthetic images, although it shows a significant improvement as compared to the Zero order error, indicating a relative increase (respectively decrease) for positive (respectively negative) gaps in the apparent BMI of the subject.

**Table 5:** Mean absolute error (MAE) for apparent BMI of generated images, obtained from a pre-trained ResNet18 BMI regressor.

	**MAE** ↓										
**BMI gap**	**−8**	**−6**	**−4**	**−2**	**−1**	**0**	**1**	**2**	**4**	**6**	**8**
Ours	2.3_1.6_	2.0_1.5_	1.8_1.4_	1.7_1.3_	1.6_1.3_	1.5_1.2_	1.5_1.3_	1.7_1.4_	2.1_1.6_	2.3_1.7_	2.7_1.8_
Zero order	7.2_2.0_	5.6_1.9_	3.8_1.8_	2.2_1.4_	1.7_1.3_	1.5_1.2_	1.7_1.4_	2.3_1.6_	4.1_1.8_	6.0_1.8_	7.9_1.7_

Standard deviations are presented as subscripts.

**Figure 5:**
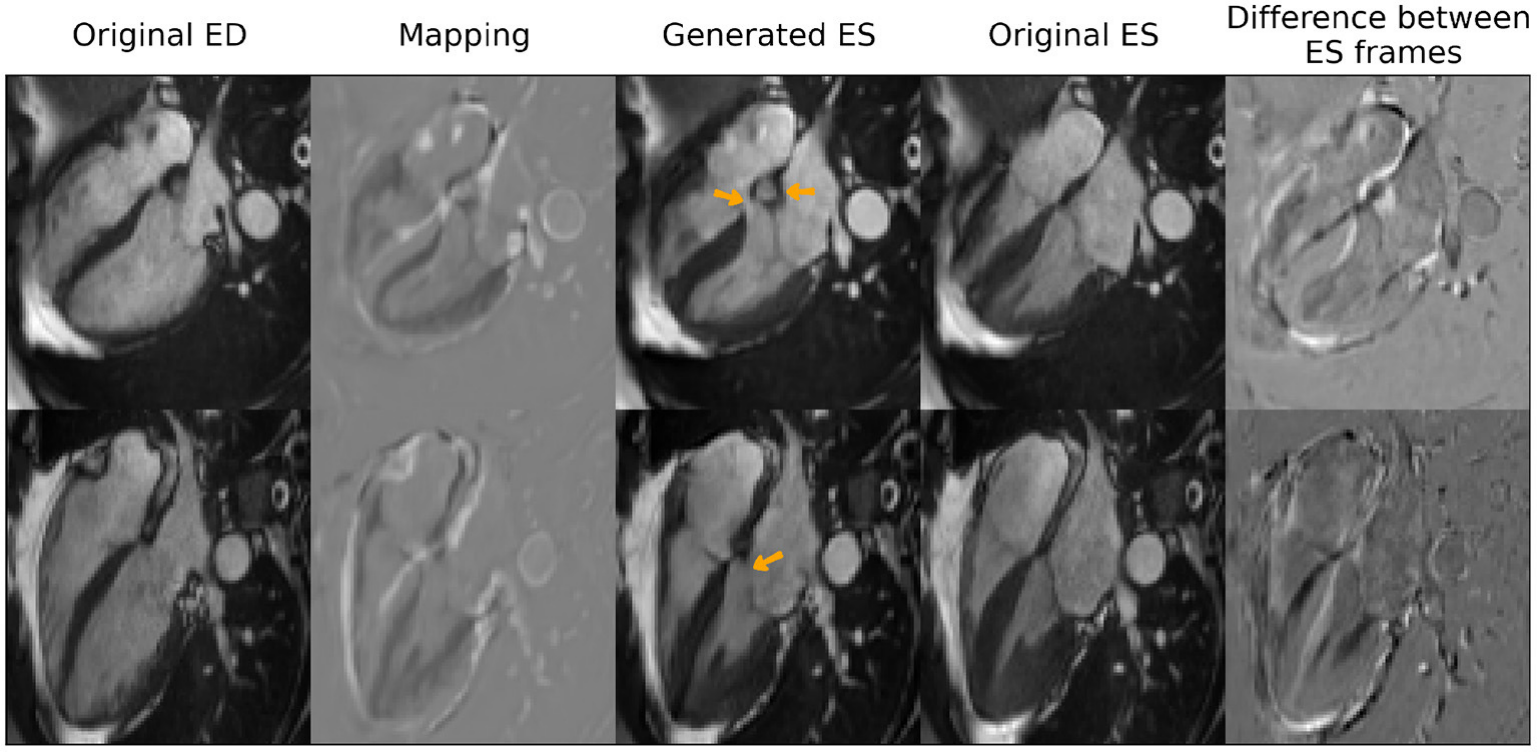
Qualitative results for end-systole (ES) frame generation from end-diastole (ED) frames. Orange arrows highlight clinical inaccuracies of the generated images such as incomplete interventricular septum or mitral valve or an extra “blob” in between the atria. Reproduced by kind permission of UK Biobank ©.

With regards to the transformation of cardiac time frames, the model obtained a root mean square error between generated images and the real ES frames of 0.06 (±0.01), when compared at the whole image level. Figure 5 shows some qualitative results obtained for this task that include the generated mapping and the pixel-wise absolute difference between the generated frames and the real ones. As observed in the figure, the model captured the thickening of the myocardium, the contraction of the right ventricle as well as the smaller changes in size in the atria between ED and ES. However, several hallucinations were also introduced (highlighted with orange arrows) by the model that are not clinically accurate.

## Discussion

A conditional generative model is proposed that allows for the modification of a cardiac image in two directions, i.e., for increased and decreased age. This is the first approach, to the best of our knowledge, for modeling the aging heart trained only on cross-sectional data. Realistic modifications are obtained without the need of complicated pre-processing steps, such as image registration or histogram matching, or of manual subdivision of the dataset in age groups. The accuracy and image quality of the results is comparable to state-of-the-art GAN methods, such as StarGAN-v2 (30), while the current model allows for a controllable target age and does not need to train several models for the aging task.

The results obtained for increasing age show in general a qualitative thickening of the interventricular septum, with the associated reduction of the LV cavity size, and an enlargement of the aorta. These changes are observed in the opposite direction for rejuvenated hearts. Quantitatively, there is a clear tendency for reduced LV size with age that is consistent with the literature (1, 2). The interventricular septum average width however, is reduced for both increased and decreased age, with the literature signaling this region as the most affected by the asymmetrical concentric LV hypertrophy observed with age (2). The ejection fraction suffers a small decrease in the mean value, while the literature states that it is preserved with age (1), and the distribution becomes wider with age, which might be related to a potential larger group of pathological subjects for increased age and the introduction of uncontrolled bias in the model which should be investigated in future works. Finally, an increased diameter is also observed in the aorta with increased age in both sexes according to the literature (33). Notably, the aorta and the interventricular septum are important areas also for age predictors based on deep learning, according to a recent study (34).

A potential application for this method has been showcased by counter-balancing biased datasets which improves the accuracy of age regression models trained on them. Recent works in the literature (35, 36) also demonstrate the feasibility of synthetic data augmentation. Such augmentation may be especially interesting for counter-balancing datasets with an age bias between healthy and diseased patients or when there are simply not enough control subjects.

Finally, two alternative tasks are presented to demonstrate the model ability to synthesize images given cross-sectional data. On one hand, the model was able to successfully increase and decrease the apparent BMI of subjects in an analogous manner to the aging task. On the other hand, four chamber images in the ES cardiac frame were synthesized from ED frames with a relatively low error when compared to the real frame.

## Limitations

The proposed model presents several limitations. First of all, the model has not been validated against real longitudinal data. This validation is particularly challenging, since repeated visits may have images acquired at slightly different slice positions which may then introduce changes in the heart morphology not associated with age. Additionally, the time gap between visits needs to be sufficiently large in order to observe visible changes, while current longitudinal datasets have a time span of < 10 years between scans.

Secondly, the model is observed to produce images with clinical inaccuracies, as observed in Figure 5, where the synthetic images present an incomplete interventricular septum, an extra “blob” in between the right and left atria or a partially missing mitral valve. One possible approach to avoid incomplete structures is to use deformable maps, instead of modifying directly the pixel intensities, at the expense of preventing the appearance of new structures that are not present in the original image in the first place.

## Conclusions

This work proposes a conditional generative model to extract longitudinal patterns using only cross-sectional data. Such a model may be applied to compare population groups, such as subjects following a specific treatment vs. a control group, that are spread in time in a cross-sectional dataset, without the need of acquiring a cost- and time-expensive longitudinal dataset. Moreover, we demonstrate the feasibility of using the generated images for dataset balancing.

## Data availability statement

The code used in this study is available at the following link: https://github.com/vicmancr/CardiacAging. The data used in this study belongs to the UK Biobank initative and is available after a successful application process at https://www.ukbiobank.ac.uk/. The data is not open-sourced. It belongs to the UK Biobank intiative. They are the data holders.

## Ethics statement

Ethical review and approval was not required for this study in accordance with the local legislation and institutional requirements. Written informed consent was not required for this study in accordance with the local legislation and institutional requirements.

## Author contributions

VC, TX, XL, PS, ST, SS, and KL contributed to the conceptualization of this work. VC, TX, XL, PS, CM-I, and SS designed the methodology. VC developed the software tools necessary for preprocessing and analysing image files and for training the models. All authors reviewed the manuscript. All authors contributed to the article and approved the submitted version.

## Funding

This research has been conducted using the UK Biobank Resource under Application Number 2964. This work was partly funded by the European Union's Horizon 2020 research and innovation program under grant agreement number 825903 (euCanSHare project). SP acknowledges the British Heart Foundation for funding the manual analysis to create a cardiovascular magnetic resonance imaging reference standard for the UK Biobank imaging resource in 5000 CMR scans (www.bhf.org.uk; PG/14/89/31194). SP acknowledges support from the National Institute for Health Research (NIHR) Biomedical Research Centre at Barts. SP acknowledges support from and from the SmartHeart EPSRC programme grant (www.nihr.ac.uk; EP/P001009/1). This article was supported by the London Medical Imaging and Artificial Intelligence Centre for Value Based Healthcare (AI4VBH). This work was supported by Health Data Research UK. ST acknowledges the support of Canon Medical and the Royal Academy of Engineering and the Research Chairs and Senior Research Fellowships scheme (grant RCSRF1819\8\25).

## Conflict of interest

The authors declare that the research was conducted in the absence of any commercial or financial relationships that could be construed as a potential conflict of interest.

## Publisher's note

All claims expressed in this article are solely those of the authors and do not necessarily represent those of their affiliated organizations, or those of the publisher, the editors and the reviewers. Any product that may be evaluated in this article, or claim that may be made by its manufacturer, is not guaranteed or endorsed by the publisher.
