# Supplementary material for: Cardiac aging synthesis from cross-sectional data with conditional generative adversarial networks
Source: Front Cardiovasc Med. 2022 Sep 23;9:983091. doi: 10.3389/fcvm.2022.983091 (PMC9537599; doi:10.3389/fcvm.2022.983091)
Supplement: Supplementary file 1 [file Data_Sheet_1.pdf]

SUPPLEMENTARY MATERIAL

Segmentation model

A segmentation model based on a classical U-Net architecture (Ronneberger et al., 2015) was used in this work. Binary cross entropy was considered as loss function and several data augmentation methods were used to increase the generalizability of the model, namely random bias field perturbation, random contrast adjustment, random histogram shifting, small random rotations up to 15 degrees and small elastic deformations. The model was trained for 100 epochs and the resulting weights were used to generate automatic annotations for all other participants in this work and for synthesized images, as will be explained later. The prediction step included post-processing steps for refining the final annotation, namely a step to fill holes surrounded by pixels from a single class and a selection of the largest connected component for each class.

Adam optimizer was used with a learning rate of 10^{-4} and first and second moments equal to 0.9 and 0.999, respectively. A batch size of 16 images with shapes 128^2 was used. The model was run on a Nvidia 3090 GPU card and took 30 minutes to train. The final validation average Dice score over all classes was 98.9%.

Sex classifier for Fréchet inception distance computation

An InceptionV3 model (Szegedy et al., 2016) was pre-trained to classify sex on the training subset of 15k subjects and later used for computing Fréchet inception distance (FID) between synthesized and real images. Binary cross entropy was used as loss function. The image size was set to 299^2, following the original implementation of InceptionV3 model. The data augmentation transformations considered were random bias field addition, random histogram shift and random contrast adjustment. Adam optimizer was used with a learning rate of 10^{-4} and first and second moments equal to 0.9 and 0.999, respectively. The batch size corresponded to 16 images. The model was run for 100 epochs and took 12 hours to completion. The final classification accuracy on the validation set was 99.0%.

The features for computing FID were extracted for the first layer block that resulted in 2048 variables per image.

Age regressor

A ResNet18 (He et al., 2016) model was trained on the training subset of 14,788 subjects to predict age from an input image. The setting and data augmentation used for training was the same as for the sex classifier. The image size was 128^2 to match the shape of the synthesized images. The model was run for 100 epochs and took 8 hours to completion with a batch size of 8 images.
